# Cost-effectiveness of Chloride-liberal versus Chloriderestrictive Intravenous Fluids among Patients Hospitalized in the United States

**DOI:** 10.36469/9829

**Published:** 2016-04-07

**Authors:** Louise Perrault, Dilip Makhija, Idal Beer, Suzanne Laplante, Sergio Iannazzo, Karthik Raghunathan

**Affiliations:** 1 International Market Access Consulting, Inc., Zug, Switzerland; 2 Baxter Healthcare Corporation, Deerfield, IL, USA; 3 SIHS Health Economics Consulting, Torino, Italy; 4 Duke University Medical Center, Division of Veterans Affairs, Durham, NC, USA

**Keywords:** intravenous fluid therapy, crystalloids, plasma substitutes, electrolytes, intravenous rehydration solutions, cost-effectiveness analysis

## Abstract

**Background:** Patients developing acute kidney injury (AKI) during critical illness or major surgery are at risk for renal sequelae such as costly and invasive acute renal replacement therapy (RRT) and chronic dialysis (CD). Rates of renal injury may be reduced with use of chloride-restrictive intravenous (IV) resuscitation fluids instead of chloride-liberal fluids.

**Objectives:** To compare the cost-effectiveness of chloride-restrictive versus chloride-liberal crystalloid fluids used during fluid resuscitation or for the maintenance of hydration among patients hospitalized in the US for critical illnesses or major surgery.

**Methods:** Clinical outcomes and costs for a simulated patient cohort (starting age 60 years) receiving either chloride-restrictive or chloride-liberal crystalloids were estimated using a decision tree for the first 90-day period after IV fluid initiation followed by a Markov model over the remainder of the cohort lifespan. Outcomes modeled in the decision tree were AKI development, recovery from AKI, progression to acute RRT, progression to CD, and death. Health states included in the Markov model were dialysis free without prior AKI, dialysis-free following AKI, CD, and death. Estimates of clinical parameters were taken from a recent meta-analysis, other published studies, and the US Renal Data System. Direct healthcare costs (in 2015 USD) were included for IV fluids, RRT, and CD. US-normalized health-state utilities were used to calculate quality-adjusted life years (QALYs).

**Results:** In the cohort of 100 patients, AKI was predicted to develop in the first 90 days in 36 patients receiving chloride-liberal crystalloids versus 22 receiving chloride-restrictive crystalloids. Higher costs of chloride-restrictive crystalloids were offset by savings from avoided renal adverse events. Chloride-liberal crystalloids were dominant over chloride-restrictive crystalloids, gaining 93.5 life-years and 81.4 QALYs while saving $298 576 over the cohort lifespan. One-way sensitivity analyses indicated results were most sensitive to the relative risk for AKI development and relatively insensitive to fluid cost. In probabilistic sensitivity analyses with 1000 iterations, chloride-restrictive crystalloids were dominant in 94.7% of iterations, with incremental cost-effectiveness ratios below $50 000/QALY in 99.6%.

**Conclusions:** This analysis predicts improved patient survival and fewer renal complications with chloriderestrictive IV fluids, yielding net savings versus chloride-liberal fluids. Results require confirmation in adequately powered head-to-head randomized trials.

## BACKGROUND

Acute kidney injury (AKI) is a serious complication of critical illness and major surgery, and is associated with short- and long-term impacts on both morbidity and mortality.[1-3] The incidence of AKI is increasing rapidly as the burden of critical illness and major surgery increases worldwide and, as associated mortality rates decrease, more patients face long-term sequelae of AKI.[4] A recent systematic review and meta-analysis of outcomes in patients with AKI, which included 13 retrospective cohort studies involving approximately 1.5 million patients, concluded that AKI is an independent risk factor for developing incident chronic kidney disease (CKD), progressive CKD, and end-stage renal disease (ESRD).[5] As reported in large observational studies, renal sequelae of AKI require treatment with costly and invasive renal replacement therapy (RRT) and are associated with significantly increased healthcare resource utilization and costs.[6-8] Avoiding AKI may, therefore, reduce its clinical and economic burden following critical illness and major surgery, particularly among patients with existing CKD at baseline.

The potential influence of intravenous (IV) crystalloid choice, during resuscitation in the critical care and perioperative setting, on modifying the risk of developing AKI was the subject of a recent meta-analysis.[9] Recommendations support therapy with IV crystalloids during resuscitation in critical illness, trauma and major operations,[10-12] as well as during severe sepsis and septic shock.[13] An ideal IV fluid should improve circulation when used for resuscitation without concurrent serious or frequent adverse effects, and should also be inexpensive and widely available.[14] Physiologically balanced IV crystalloid solutions contain cations and anions in concentrations closer to normal human plasma whereas some other commonly used IV crystalloid solutions like isotonic 0.9% saline contain significantly higher chloride concentrations.[14] Physiologically balanced IV crystalloids achieve electrical neutrality with anions other than chloride such as lactate, acetate, or gluconate, and also have a strong ion difference similar to human plasma. These anions are metabolized rapidly, thereby avoiding the metabolic acidosis that can accompany a decline in strong ion difference as is caused by chloride-liberal IV fluid therapy.[14]

Small randomized controlled trials (RCTs) and several large observational studies provide some evidence that renal injury may be decreased with chloride-restrictive (balanced) crystalloid solutions versus chlorideliberal solutions.[9] In healthy volunteers, infusions of 0.9% saline result in demonstrable reductions of renal blood flow and renal cortical tissue perfusion, effects that are not seen with balanced solutions.[15] Hospitalized patients receiving chloride-restrictive crystalloids have been reported to have lower rates of AKI and RRT.[16-18] A recent meta-analysis including 21 studies comprising 6253 patients receiving chloride-restrictive versus chloride-liberal IV fluids in perioperative or critical care settings reported that the use of chlorideliberal fluids was associated with a significantly higher risk of AKI (relative risk [RR] 1.64, 95% confidence interval [CI] 1.27, 2.13; P<0.001).[9] In a large risk-adjusted study of patients with systemic inflammatory response syndrome receiving chloride-restrictive solutions versus isotonic 0.9% saline (or other chlorideliberal fluids), recipients of chloride-restrictive solutions had lower in-hospital mortality, shorter length of stay, fewer readmissions, and fewer complications (cardiac, infectious, coagulopathy). However, economic consequences were not estimated.[19] A cost-minimization analysis suggested possible savings with the use of a balanced crystalloid as a resuscitation fluid for critically ill trauma patients. However, this analysis was based solely on a hospital's short-term perspective (expenses related to the IV fluids themselves, electrolyte replacement, and nurse labor costs), without considering the long-term economic impact of crystalloid choice.[20]

Data on the economic consequences of crystalloid choice are limited but are important because exposure to IV crystalloids is universal in perioperative and critical care setting. Furthermore, there is a clear contrast (based on chloride content) between available choices and important clinical outcomes—rates of renal injury—may differ with meaningful economic implications at the population level. We therefore address this knowledge gap by comparing the cost-effectiveness of chloride-restrictive versus chloride-liberal crystalloids used during fluid resuscitation or for the maintenance of hydration among patients hospitalized for critical illnesses or major surgery.

## METHODS

### Model Structure

A cost-effectiveness model was constructed utilizing clinical estimates from a recently published metaanalysis.[9] A hybrid model design was used, consisting of a decision tree for the first 90-day period following model entry, then proceeding into a Markov cohort model as this technique is better suited to modeling long-term clinical outcomes and costs in chronic disease states.[21]

The decision tree component simulated the flow of a study cohort through two possible treatment arms: one receiving chloride-restrictive IV fluids and the other chloride-liberal IV fluids. As shown in Figure 1, two outcomes could occur: AKI or no AKI. If AKI developed, patients could then either recover or progress to acute RRT or die. Those progressing to acute RRT could either subsequently recover or require chronic dialysis or die. Patients that did not develop AKI could either remain alive without dialysis by the end of 90 days, or die.

Following the initial 90-day period, surviving patients entered the Markov component of the model, in which outcomes and costs were modeled in annual cycles for the remainder of the cohort's lifespan (i.e., Markov cycles continued until all patients had died). As presented in Figure 2, the Markov design was conditioned on four possible health states: requiring chronic dialysis, not requiring chronic dialysis without prior AKI or acute RRT, not requiring chronic dialysis with prior AKI and/or acute RRT, and death (as an absorbing state). Each patient entered the Markov model in the health state in which they exited the decision tree. In each Markov cycle patients could remain in their existing health state or die; in addition, patients not requiring dialysis (with or without prior AKI/acute RRT) could progress to chronic dialysis.

### Model Patient Population

The target population considered in the model was the patient population included in the meta-analysis: critical care patients in the United States requiring IV fluids for resuscitation, including those with acute illness, infections and/or sepsis, trauma, as well as patients undergoing major operative procedures.[9] The age of patients at cohort entry was set at 60 years, to reflect the patient population expected in the near future as the population of the United States ages. The model time period commenced in 2015, and the cohort was followed until the last patient died.

### Perspective and Discounting

The cost-effectiveness analysis adopted a US third-party payer perspective, considering only direct costs for IV resuscitation fluid solutions, acute treatment of AKI (i.e., acute RRT), and chronic dialysis. An annual discount rate of 3% was applied to both costs and health outcomes, as recommended in the current version of the Academy of Managed Care Pharmacy formulary submission guidelines.[22]

**Figure 1.** Decision Tree Structure (90-day Period Following ICU Admission)

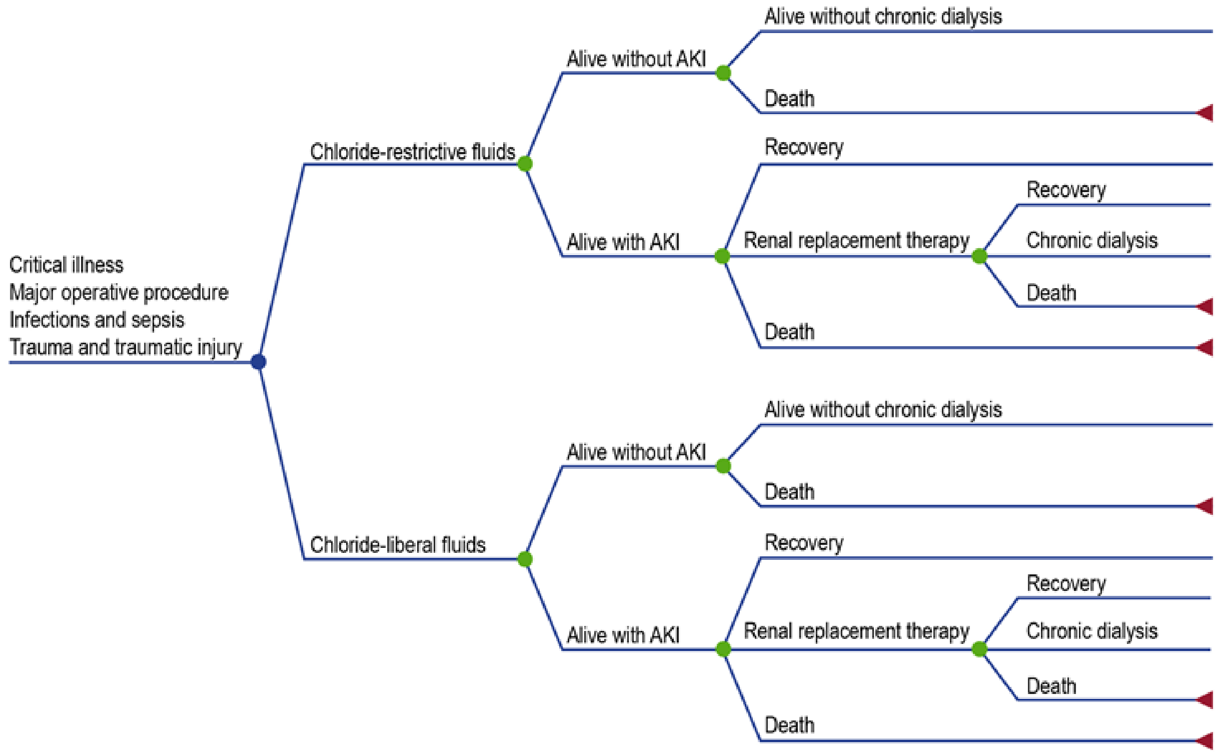

AKI: acute kidney injury; ICU: intensive care unit

**Figure 2.** Markov Model Health States (Annual Cycles)

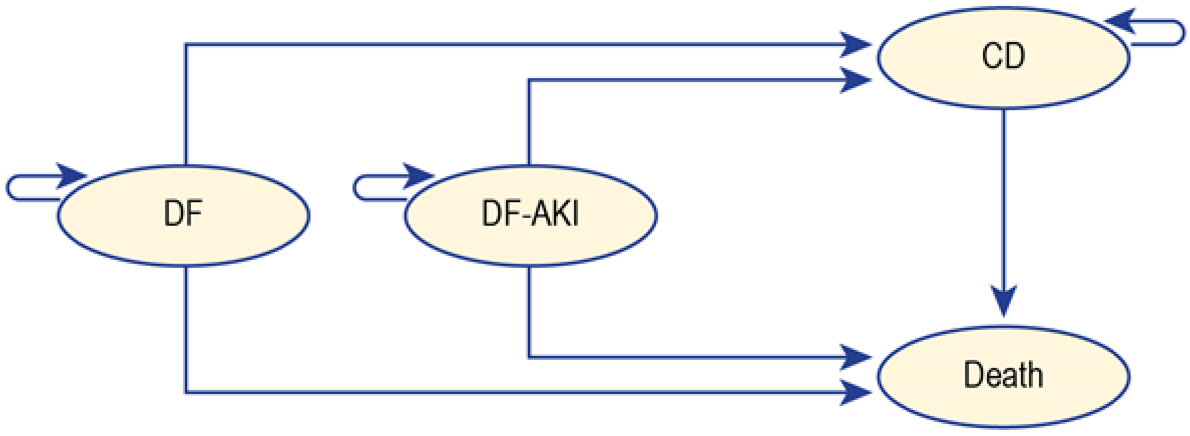

CD: chronic dialysis; DF: dialysis-free; DF-AKI: dialysis-free post-acute kidney injury/renal replacement therapy

## Comparators

This analysis compared two possible exposures: chloride-restrictive versus chloride-liberal IV crystalloid fluids. For each, fluid composition and volume of fluid infused per patient were based on the fluids administered in the prospective study reported by Yunos et al,[18] (included in the meta-analysis we used as the basis of clinical outcomes).[9] We assumed that the baseline risks of AKI are comparable between the two treatment arms, and that these IV fluids are used exclusively.

## Model Inputs

Values of the probabilities of transitioning between model health states are listed in Supplementary Table 1. For the decision tree, the probability of developing AKI in the first 90 days in patients receiving chloride-restrictive IV fluids was derived from a retrospective, observational study of records from Veterans Affairs (VA) intensive care units (ICUs).[23] The RR for developing AKI with chloride-restrictive versus chloride liberal fluids was obtained from the recent meta-analysis and was used to calculate the probability of developing AKI among patients receiving chloride-liberal rather than chloride-restrictive IV fluids.[9] The mortality rate in patients not developing AKI during the 90-day period was estimated based on data from the control group in the Finnish Acute Kidney Injury (FINNAKI) study.[24] Probabilities of transitioning from AKI to recovery, or acute RRT, or death were derived from the VA ICU study.[23] Probabilities of recovery, progression to chronic dialysis, and death following acute RRT were based on results of the Randomized Evaluation of Normal versus Augmented Level (RENAL) Replacement Therapy Study.[25]

For the Markov model, the per-cycle mortality probability in patients exiting the decision tree without experiencing AKI or dialysis was calculated from life tables for the United States in 2009.[26] The probability in this patient subgroup of transitioning to chronic dialysis was estimated from the incidence of reported ESRD compiled by the US Renal Data System.[27] In dialysis-free patients with prior AKI (either with or without acute RRT) in the 90-day period, the probabilities of progression to chronic dialysis and to death were calculated from long-term follow-up data from the RENAL study.[28] The probability of death among patients on chronic dialysis was synthesized from data from the US Renal Data System.[27]

The overall impact of chronic dialysis progression, both in survivors of AKI and in patients without AKI exposed to chloride-restrictive versus chloride liberal crystalloids was extrapolated over the model's time horizon (i.e., the cohort lifetime). Parametric curve fitting was applied to extrapolate survival and progression to dialysis beyond the duration of observed data.

## Health Outcomes

Health outcomes considered in the analysis were overall survival (OS) measured in life-years (LY), and survival adjusted for health-related quality of life (HRQoL). To calculate quality-adjusted life-years (QALYs), LYs were multiplied by health-state utilities. In the absence of utilities derived specifically for all health states incorporated in the model, two health utilities were applied. Patients not on chronic dialysis, whether or not they previously had AKI, were assigned a utility of 0.86, based on age-adjusted US norm-based scores on the five-dimension EuroQol health status questionnaire (EQ-5D) for individuals 65–74 years old, as reported in the National Health Measurement Study.[29] Patients on chronic dialysis, whether or not they previously had AKI, were assigned a utility score of 0.56, as reported in a meta-analysis of preference-based HRQoL valuations for patients on RRT.[30]

### Costs

All costs are reported in US dollars for the year 2015; cost data from other years were inflated to 2015 using the Medical Care Services component of the Consumer Price Index. The costs of a 72-hour infusion of chloride-restrictive and chloride liberal crystalloids were set at $59.28 and $38.23, respectively, based on average wholesale prices of fluids available in the US and average chloride-restrictive and chloride-liberal fluid composition and volumes reported in the study by Yunos et al.,[18] calculated as shown in Supplementary Table 2. The 90-day cost of acute RRT was set at $6310.05 (for 7-day treatment), based on a recent decision analysis that compared acute intermittent and continuous RRT.[31] The 90-day cost of chronic dialysis was $22 161.60, based on data from the US Renal Data System.[32]

### Cost-effectiveness Ratios

Incremental cost-effectiveness ratios (ICERs) are presented for both cost per LY gained and cost per QALY gained.

### Sensitivity Analyses

To assess the sensitivity of model results to variation in the underlying parameters, a series of one-way sensitivity analyses were conducted with parameters varied above and below the base-case values reported above by the standard deviation (SD) or 95% CI when these were available, or ± 10% if the SD and 95% CI were unavailable. In addition, discount rates were tested in the 0% to 10% range. The upper- and lower-bound values used in these one-way sensitivity analyses are reported in Supplementary Table 1. Results of these sensitivity analyses are presented as net monetary benefit (NMB), an alternate method of handling uncertainty in cost-effectiveness analysis, in which incremental cost is subtracted from the incremental effect valued in dollars.[33] For the sensitivity analyses presented here, NMB is calculated assuming a willingness to pay $50,000 per QALY gained—this is the bottom end of the range of ICER thresholds commonly considered to represent acceptable cost-effectiveness in the United States: $50 000–100 000/QALY.[34]

In addition, to address the uncertainty in the parameters used in the model, a distribution-based probabilistic sensitivity analysis was conducted. The parameters included in this sensitivity analysis and their corresponding distributions are shown in Supplementary Table 1. The probabilistic sensitivity analysis was performed over 1000 iterations.

## RESULTS

### Base-case Analysis

Out of a total cohort of 100 patients with either critical illness or undergoing major surgery, AKI was predicted to develop in the first 90 days in 36 patients receiving chloride-liberal IV crystalloids compared with 22 receiving chloride-restrictive IV crystalloids. Over the long term, more patients receiving chloride-restrictive fluids were predicted to remain alive and not on chronic dialysis, with mortality rates converging for the two groups only at the end of the cohort lifespan due to age-related mortality (Figure 3).

As shown in Table 1, patients receiving chloride-restrictive IV crystalloids were predicted to have higher overall survival rates than those receiving chloride-liberal IV crystalloids, yielding a relative gain of 93.5 LYs per 100 patients over the cohort lifespan. Patients on chloride-restrictive fluids also accrued more QALYs

(81.4 more per 100 patients) at a lower cost (savings of $298 576 per 100 patients) when compared with chloride-liberal fluids. Thus, in the base-case analysis, chloride-restrictive crystalloids were dominant in terms of both cost per LY and cost per QALY gained.

**Table 1.** Base-case* Cost-effectiveness Results (per 100 patients)

| | Chloride-restrictive Fluid | Chloride-liberal Fluid | Difference |
|---|---|---|---|
| Outcomes | | | |
| LY | 1174.4 | 1080.9 | 93.5 |
| QALY | 1006.9 | 925.6 | 81.4 |
| Costs, $ | | | |
| Fluids | $5928 | $3823 | $2105 |
| Initial 90-day direct costs | $7287 | $11 951 | -$4664 |
| Long-term direct costs | $1 006 583 | $1 302 600 | -$296 017 |
| Total | $1 019 798 | $1 318 374 | -$298 576 |
| ICER | | | |
| $/LY | | | Dominant |
| $/QALY | | | Dominant |

*Age 60 years at cohort entry; 3% annual discounting of costs and outcomes over lifetime horizon. ICER: incremental cost-effectiveness ratio; LY: life-year; QALY: quality-adjusted life-year

**Figure 3.** Health-state Outcomes in the Model Cohort

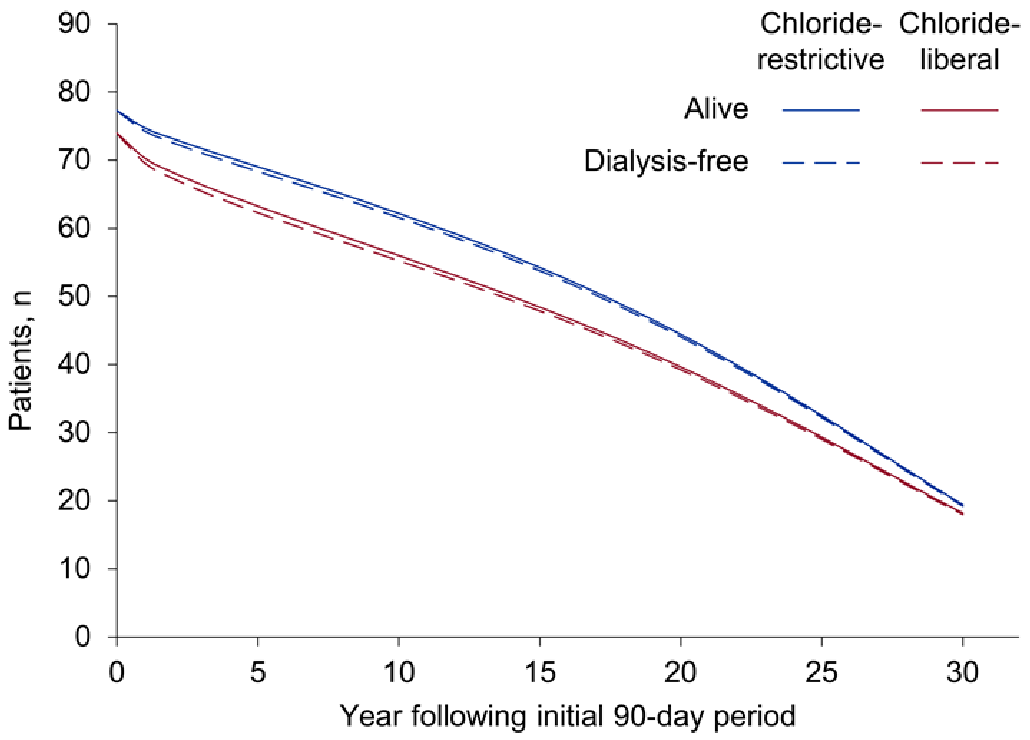

Note: x-axis scale refers to years within the Markov model (i.e., Year 0 begins at the end of the 90-day initial period modeled in the decision tree).

The cost savings with chloride-restrictive IV fluids were attributable to lower costs from decreased renal adverse effects, which offset the increased direct costs for these fluids. Notably, the higher direct cost of chloride-restrictive IV fluids was offset more than two-fold within the first 90-day period by lower costs for acute RRT and chronic dialysis.

### Sensitivity Analyses

In the one-way sensitivity analyses, the five model parameters that had greatest influence on the NMB results were: the RR for AKI associated with chloride-liberal versus chloride-restrictive IV fluids, the age of the cohort at model entry, the annual discount rate for benefits, the probability of remaining alive with AKI in the chloride-restrictive arm of the decision tree, and the probability of remaining alive without AKI in the decision tree (Figure 4). However, in the one-way sensitivity analyses for these and all other model parameters, the NMB results were always well below the upper bound of the conventional range for willingness-to-pay thresholds in the United States, namely $100 000/QALY.[34] Variation in the cost of chloride-restrictive and chloride-liberal IV fluids had only a minor impact on the results.

**Figure 4.** One-way Sensitivity Analyses of Net Monetary Benefit

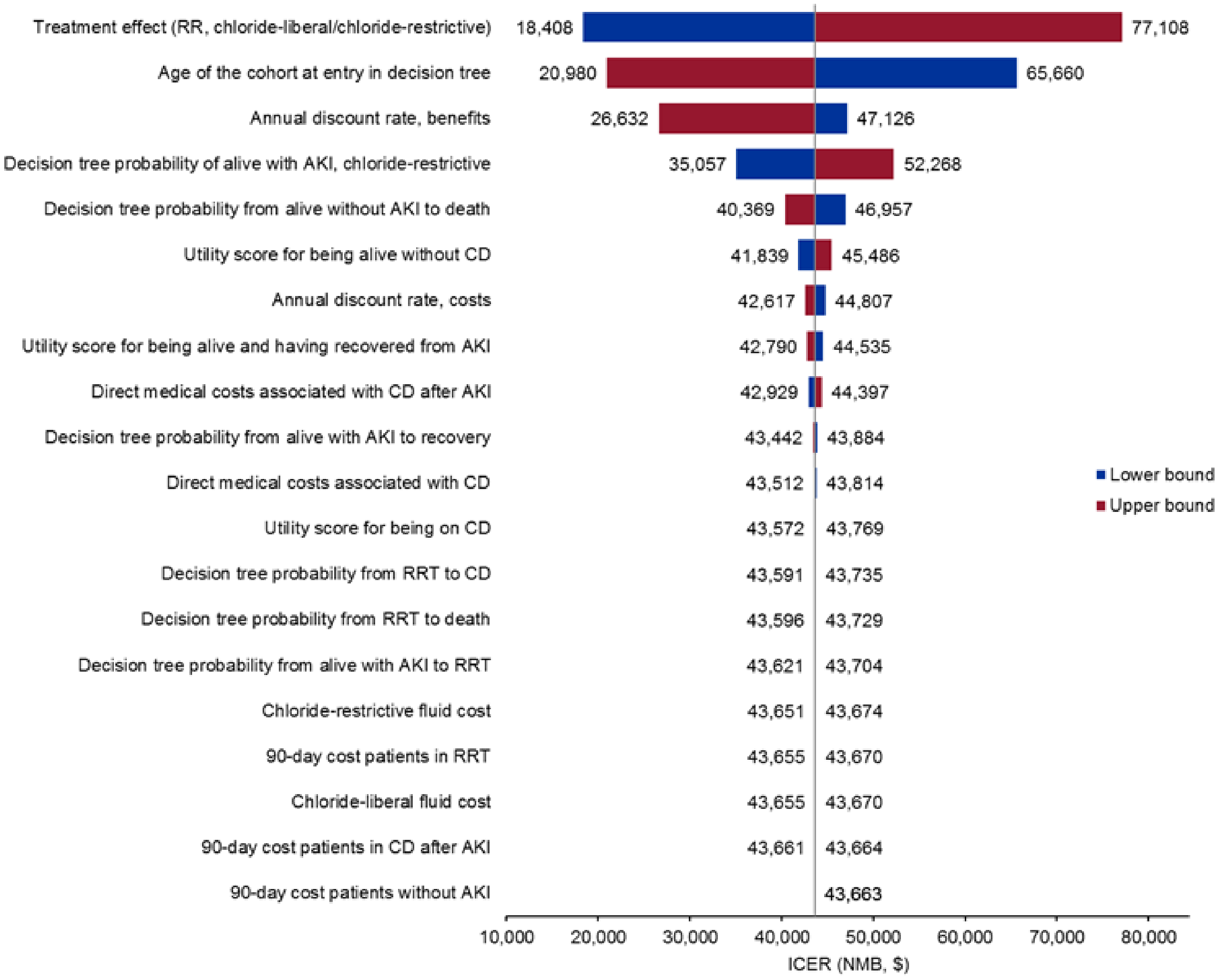

AKI: acute kidney injury CD; chronic dialysis; ICER: incremental cost-effectiveness ratio; NMB: net monetary benefit; RR: relative risk; RRT: renal replacement therapy

In the probabilistic sensitivity analysis, chloride-restrictive crystalloids were dominant in 94.7% of iterations (Figure 5). Furthermore, ICERs were below \$50 000/QALY in 99.6% of iterations, and below \$100 000/QALY in 99.8% of iterations.

**Figure 5.** Probabilistic Sensitivity Analysis of Cost per QALY (1000 Iterations)

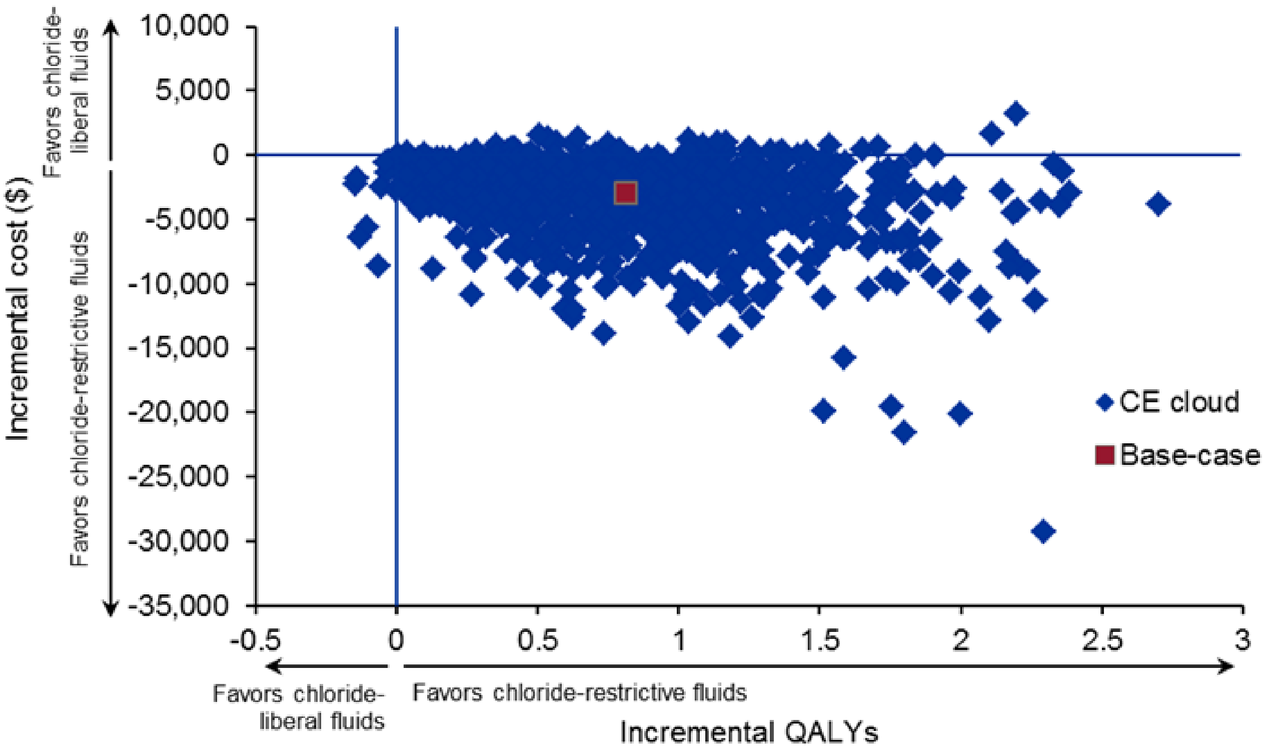

CE: cost-effectiveness; QALY: quality-adjusted life-year

## DISCUSSION

We present an analysis of the cost-effectiveness of different IV crystalloids (with differences in their chloride content) among patients requiring IV fluid therapy for resuscitation or the maintenance of hydration in perioperative and critical care settings. Chloride-restrictive crystalloids were superior to chloride-liberal crystalloids and the increase in acquisition costs (associated with chloride-restrictive IV fluids) was offset by more than two-fold savings from avoiding adverse renal outcomes in the initial 90-day period, and by more than two orders of magnitude in lifetime savings. Overall, net savings with use of chloride-restrictive fluids for patients with critical illnesses or undergoing major surgery were considerable. Our results suggest that improved renal outcomes translate into reduced short- and long-term costs, highlighting the importance of IV crystalloid choice in these populations. This is particularly relevant since the incidence of AKI and associated healthcare resource utilization and costs appears to be increasing in developed countries,[4, 35] though this trend is not consistent in all countries.[36]

A previous cost-minimization analysis had compared costs among adult trauma patients receiving either a balanced crystalloid solution or 0.9% saline during the first 24 hours after traumatic injury.[20] The receipt of balanced crystalloid solutions was associated with savings of \$12.35 per patient, based solely on a reduction in the utilization of magnesium replacement when taking into account the costs of consumable supplies and nurse labor.[20] We did not include this relatively minor cost (of magnesium repletion) in our cost-effectiveness model, focusing instead on expenditures for renal replacement therapies (short and long term). Inclusion of costs for magnesium replacement in our analysis would further decrease costs in favor of chloride-restrictive fluids.

Our cost-effectiveness analysis did not focus on any specific formulation of IV crystalloids. Since not all available chloride-restrictive crystalloids are equivalent, additional research is needed to identify pharmacoeconomic differences among commercially available chloride-restrictive crystalloids (taking into account potential implications of differences in the duration of mechanical ventilation). Commonly used chloride-restrictive crystalloids differ in their concentration of electrolytes (i.e., Na, K, Cl, Ca, Mg), type of anion, pH, and osmolality, as well as their propensity to result in coagulation if co-administered with citrate-containing blood products (frequent in acute care and perioperative settings).[14] In a prospective observational study using patient-level data contained in Electronic Health Records, cardiac surgery patients receiving either Plasma-Lyte® or Normosol® had reduced rates of respiratory failure and lower daily per-patient costs versus Lactated Ringer's solution.[37] There are no large RCTs that have directly compared crystalloids based on their chloride-content in a manner that is reflective of practice in at-risk patient populations.[10,38] The recently published multicenter, cluster-randomized, double-crossover feasibility study comparing a balanced crystalloid fluid versus 0.9% saline—the SPLIT study—found no significant differences in the rates of AKI, acute RRT, or mortality, either in-hospital or in 90-day follow-up.[39] However, the SPLIT study population (patients in four different ICUs with both medical and surgical illnesses) was heterogeneous with a low overall incidence of AKI, receipt of relatively limited quantities of IV fluids (2L on average of either chloride-liberal or restrictive fluids), and with the use of Plasma-Lyte® as the non-study default crystalloid used during resuscitation. In contrast to the four ICUs included in the SPLIT study, 0.9% saline is the primary crystalloid used for resuscitation worldwide and in large quantities. A definitive study will therefore need to compare commonly used crystalloids in higher-risk populations that receive larger fluid volumes.

Our study has a number of strengths. The combination of decision-tree and Markov model allowed exploration of costs and outcomes over different (clinically relevant) time periods, allowing assessment of both the short- and long-term association between AKI and increased costs. Sensitivity analyses demonstrated that the cost-effectiveness results were generally robust to variation in model parameters, and were essentially insensitive to varying the acquisition costs for IV fluids. The key treatment effect driving differences in outcomes between the two model arms—relative risk for AKI—was derived from the most recent meta-analysis currently available,[9] increasing confidence in the applicability of these findings to current clinical practice.

This study is also subject to some limitations. It is possible that confounding factors could have influenced the association between AKI and IV fluids in the source studies that were the basis for the clinical estimates in the model. For example, in an extension of the study by Yunos et al,[18] the authors controlled for several patient characteristics but found in sensitivity analyses that other unknown confounders could have also influenced the incidence of AKI, although the finding of a significantly greater risk of AKI and RRT use with chloride-liberal IV fluids was unaltered.[17] The meta-analysis we used as the source for the clinical estimates in our model included an appropriate assessment of risk of bias in study selection,[9] but it could not control for confounding factors at the individual patient level since it incorporated study-level results reported in the primary publications. Although it is similarly not possible for us to assess potential confounding at the patient level, the results of our probabilistic sensitivity analysis help to increase confidence in our results, since our main finding that chloride-restrictive fluids were dominant was replicated in approximately 95% of iterations.

Other study limitations include the fact that only direct medical costs were considered, namely costs for the IV resuscitation fluids administered and for management of renal-related complications. There could be additional differential health effects not accounted for in the present analysis that may be associated with the chloride content of different fluids, such as cardiovascular, respiratory, gastrointestinal, neurologic, and infection-related complications. Although previous research has demonstrated increasing length of stay, mortality and costs with increasing AKI severity,[40] different levels of AKI severity were not modelled. Our calculations are based on several assumptions from diverse populations—including US,[23] Finnish,[24] and Australasian.[25] Finally, as health-state–specific utility data were not available for every model health state, only two utility scores were incorporated in the model, potentially over-simplifying the HRQoL impact of different health states.

## CONCLUSIONS

This cost-effectiveness analysis supports the value of chloride-restrictive IV fluids (crystalloid solutions with a chloride concentration <110 mEq/L) in terms of improved patient survival and fewer renal complications at a lower cost when compared with chloride-liberal fluids. Higher initial acquisition costs of chloride-restrictive IV fluids were offset several-fold by short- and long-term savings from avoiding renal adverse events. These results need to be confirmed in adequate head-to-head RCTs.

## ACKNOWLEDGEMENTS

Editorial assistance in the preparation of this manuscript was provided by W. Mark Roberts, PhD, Montreal, Canada, and was funded by Baxter Healthcare Corporation.

### Conflict of Interest Declaration

This study was sponsored by Baxter Healthcare Corporation. DM, IB, and SL are employees and shareholders of Baxter Healthcare Corporation. The development of the model was supported by Baxter Healthcare Corporation through consulting contracts with International Market Access Consulting, Inc., a company owned and directed by LP, and with SIHS SRL, a company owned and directed by SI. KR was Principal Investigator on an investigator-initiated trial supported by Baxter.

## Figures and Tables

**Figure:** Supplementary Content

